# Treatment Outcomes of Re-irradiation in Locoregionally Recurrent Rectal Cancer and Clinical Significance of Proper Patient Selection

**DOI:** 10.3389/fonc.2019.00529

**Published:** 2019-06-19

**Authors:** Seung Yeun Chung, Woong Sub Koom, Ki Chang Keum, Jee Suk Chang, Sang Joon Shin, Joong Bae Ahn, Byung Soh Min, Kang Young Lee, Nam Kyu Kim, Hong In Yoon

**Affiliations:** ^1^Department of Radiation Oncology, Yonsei Cancer Center, Yonsei University College of Medicine, Seoul, South Korea; ^2^Department of Internal Medicine, Yonsei Cancer Center, Yonsei University College of Medicine, Seoul, South Korea; ^3^Department of Surgery, Yonsei Cancer Center, Yonsei University College of Medicine, Seoul, South Korea

**Keywords:** re-irradiation, rectal cancer, locoregional recurrence, acute toxicity, late toxicity

## Abstract

**Background and Purpose:** Majority of patients with locoregionally recurrent rectal cancer will require re-irradation (reRT). This study aimed to analyze the treatment outcomes, particularly infield progression, and severe late toxicity rates after reRT for recurrent rectal cancer and further identify a subgroup of patients who may optimally benefit from reRT.

**Materials and Methods:** Patients with rectal cancer who underwent reRT to the pelvis between January 2000 and December 2017 were included for analysis.

**Results:** The records of 41 patients were retrospectively reviewed. The median follow-up period after reRT was 53.7 months (range 3.5–130.3 months). The 2-year infield progression-free rate (IPFR) was 49.4%. The 2-year overall survival (OS) and progression-free survival (PFS) rates were 55.3 and 28.5%, respectively. Severe late toxicity events occurred in 17 patients, and the median time from reRT to severe late toxicity event was 10.5 months (range 2.3–33.3 months). The 2-year severe late toxicity free-rate (SLTFR) was 55.5%, and the median SLTFR was 33.3 months. Patients who did not experience severe late toxicity events showed a significantly higher number of recurred tumors at the posterior or lateral location compared to axial or anterior location. The selected subgroup with recurrent tumor size <3.3 cm and treated with total reRT dose of >50 Gy_ab10_ (*n* = 13) showed superior IPFR, OS, and PFS to the other patients.

**Conclusion:** ReRT was a reasonable treatment option for patients with locoregionally recurrent rectal cancer. However, severe late toxicity rates were substantially high. Thus, patients indicated for ReRT with curative dose should be selected properly according to tumor size and location.

## Introduction

Rectal cancer is among the most common malignancies, with ~39,910 new cases in the United States in 2017 (1). Treatment outcomes for rectal cancer have considerably improved in recent years owing to advances in multidisciplinary treatments including surgery, radiotherapy (RT), and chemotherapy. Particularly, neoadjuvant concurrent chemoradiotherapy (CCRT) and total mesorectal excision have contributed to a decreased rate of local recurrences (2–4). However, despite advances in treatment modalities, local recurrences still occur in 5–15% of patients, and the majority of locoregional recurrences occur within the initial RT field (5, 6). Recurrences within the pelvis are associated with increased morbidity and mortality, and surgery is the first treatment option in these cases (6, 7). However, some patients are ineligible for surgery due to poor general condition or tumor extent and location (8). In these cases, RT can help in achieving R0 resection, improving local control, or palliating the symptoms (7), and re-irradation (reRT) will be necessary in the majority of these patients since most recurrences will be within the initial RT field (5).

Several factors have to be considered in planning reRT, including disease extent, organs at risk (OARs), dose fractionation, interval from previous RT, prognosis, and general condition. However, retrospective and prospective studies on reRT are limited, and the treatment schemes, median survival, and toxicity rates vary in these studies (7, 9–14). Many studies have reported the feasibility of reRT for locoregional recurrences, including preoperative RT, definitive RT, and intraoperative RT (15–19). We have previously reported the treatment outcomes and toxicity rates of patients with recurrent rectal cancer who underwent reRT in our institution (20). However, because the study included only a small number of patients and the follow-up period was short, we wanted to perform a retrospective review with more patients and longer follow-up. In this study, we aimed to analyze the treatment outcomes, particularly infield progression, and severe late toxicity after reRT for recurrent rectal cancer and further identify a subgroup of patients who may optimally benefit from reRT without increased severe late toxicity.

## Materials and Methods

### Patient Selection

We retrospectively reviewed the medical records of patients with rectal cancer who underwent reRT to the pelvis from January 2000 to December 2017. Patients who underwent surgery and preoperative or postoperative RT with or without chemotherapy as initial curative treatment were included, while those who did not receive RT as part of initial treatment and those who did not receive reRT to the pelvis were excluded. The study was approved by the Institutional Review Board of Severance hospital (IRB no. 4-2019-0344).

### Re-Irradiation

A multidisciplinary team determined whether patients should receive reRT. All patients' imaging studies were reviewed and were clinically diagnosed as rectal cancer recurrences. Thus, pathologic diagnosis was not mandatory. The planning target volume was the recurred gross tumor volume plus 0.5–3 cm margin depending on the physician's preference. In most cases, margins were 0.5–1 cm and were reduced when OARs were close. When planning, dose-constraints for OARs were prioritized compared to target volumes in majority of cases. Median reRT dose was 50 Gy (range 30–60 Gy) with a fractional dose of median 2 Gy (range 1.2–6.0 Gy). Thirty-eight patients received reRT by conventional fractionation, two patients received reRT by hypofractionated regimen, and one patient received BID treatment. In our institution, tomotherapy was first introduced in 2006; since then, the number of patients receiving reRT via intensity-modulated radiotherapy (IMRT) increased. Prior to 2010, more than half of the patients (61.9%) received reRT via 3D conformal radiotherapy (3DCRT), but since 2010, 90% of the patients received reRT via IMRT. Hypofractionated reRT with fractional doses of 5 or 6 Gy was performed via cyberknife and conventional fractionation reRT was performed via IMRT.

As for chemotherapy, twenty-one patients received concurrent chemotherapy during reRT with regimens such as xeloda, 5-fluorouracil/leucovorin and 5-fluorouracil/leucovorin/oxaliplatin.

### Response and Toxicity Assessment and Follow-Up

Treatment response was evaluated according to the Response Evaluation Criteria in Solid Tumors. Follow-up evaluation was performed at 1 and 3 months after reRT and routinely thereafter. Acute and late toxicity events were thoroughly reviewed retrospectively and graded according to the National Cancer Institute Common Terminology Criteria for Adverse Events (CTCAE) version 4.03. Severe toxicity events were defined as toxicity events of grade 3 or higher. The primary endpoints were severe late toxicity-free rate (SLTFR) and infield progression-free rate (IPFR). Infield progression was defined as recurrence or disease progression within the re-irradiation field. The secondary endpoints were overall survival (OS), progression-free survival (PFS), and outfield progression-free rate (OPFR). Outfield progression was defined as recurrence or progression of the disease outside the re-irradiation field.

### Statistical Analysis

The cumulative probabilities of SLTFR, IPFR, OS, PFS, and OPFR were calculated using the Kaplan-Meier method and compared using log-rank test for subgroup analysis. The SLTFR, IPFR, OS, PFS, and OPFR were defined as the time from reRT until the corresponding event or the date of last follow-up in our institution. Univariate and multivariate analyses were performed using Cox's regression. A backward elimination method including all the variables was used for multivariate analyses. Clinical factors associated with severe late toxicity and infield progression were analyzed using the χ^2^ and Fisher's exact tests. All analyses were performed using the SPSS version 23.0 (IBM Inc., Armonk, NY, USA).

## Results

### Patient, Tumor, and Treatment Characteristics at Initial Diagnosis and at Recurrence

A total of 41 patients were included for analysis. The patient, tumor, and treatment characteristics at the time of initial diagnosis are shown in Supplementary Tables 1, 2. The majority of patients were male, and the median age was 55 years. More than 50% of the patients were diagnosed with low rectal cancer with definitive or borderline circumferential resection margin threatening. Half of the patients received preoperative CCRT, while the other half received postoperative RT, and most patients received adjuvant chemotherapy. All except two patients received 3DCRT, with a median dose of 50.4 Gy (range 45–60 Gy).

The patient, tumor, and treatment characteristics including patterns of failure at the time of recurrence prior to reRT are shown in Tables 1, 2. More than half of the recurrences were local only, and around 90% were local and/or regional recurrences. The median size of the recurred tumor was 3.3 cm. The majority (63.4%) of recurrences were infield recurrences within the ≥50 Gy isodose line. The location of the recurred tumor was classified using the Guillem classification criteria as follows: (1) axial: not involving the anterior, posterior, or lateral pelvic walls—including anastomotic recurrence after low anterior resection, local recurrence after transanal or transsphincteric excision, and perineal recurrence after abdomino-perineal resection; (2) anterior: involving the urinary bladder, vagina, uterus, seminal vesicles, or prostate; (3) posterior: involving the sacrum and coccyx; and (4) lateral: involving the bony pelvic sidewall or sidewall structures including the iliac vessels, pelvic ureters, lateral lymph nodes, pelvic autonomic nerves, and sidewall musculature. The numbers of recurred tumors were similar for each classification. ReRT was performed via IMRT or CyberKnife in 26 patients. Because few patients received hypofractionated RT, the range of total dose and dose per fraction for reRT varied widely. The median reRT dose was 50 Gy (range 30–60 Gy), with a median fractional dose of 2 Gy (range 1.2–6.0 Gy). The median follow-up period after reRT was 53.7 months (range 3.5–130.3 months).

**Table 1:** Patient and tumor characteristics at recurrence.

	***n***	**%**
**CEA (ng/mL)**		
Median (range)	6.82	(0.20–109.28)
**Histology**		
Adenocarcinoma	14	34.1
Mucinous ca	1	2.4
Not confirmed	26	63.4
**Pattern of failure**		
Local only	24	58.5
Regional only	6	14.6
Locoregional	6	14.6
Local & distant	2	4.9
Regional & distant	1	2.4
Local, regional & distant	2	4.9
**Tumor size (cm)**		
Median (range)	3.3	(1.5–11.0)
<3.3 cm	20	48.8
≥3.3 cm	21	51.2
**Initial RT isodose line**		
<40 Gy	3	7.3
40–45 Gy	7	17.1
45–50 Gy	5	12.2
≥ 50 Gy	26	63.4
**Guillem class**		
Axial	10	24.4
Anterior	8	19.5
Posterior	9	22
Lateral	8	19.5
Anterior & lateral	5	12.2
Posterior & lateral	1	2.4
**rT stage**		
T0	6	14.6
T1	0	0
T2	0	0
T3	7	17.1
T4	28	68.3
**rN stage**		
N0	29	70.7
N1	10	24.4
N2	2	4.9
**R-stage**		
II	26	63.4
III	10	24.4
IV	5	12.2

*CEA, carcinoembryonic antigen; RT, radiotherapy*.

**Table 2:** Treatment characteristics at recurrence.

	***n***	**%**
**RT modality**		
3DCRT	15	36.6
IMRT/Cyberknife	26	63.4
**Re-RT total dose (Gy)**		
Median (range)	50	(30.0–60.0)
**Re-RT dose per fraction (Gy)**		
Median (range)	2	(1.2–6.0)
**Re-RT total dose in EQD2 (Gy**_**ab10**_**)**		
Median (range)	50.82	(28.00–65.00)
**Re-RT total dose in EQD2 (Gy**_**ab3**_**)**		
Median (range)	51.84	(25.20–86.40)
**Sum total dose (Gy)**		
Median (range)	104	(43.2–119.4)
**Sum total dose in EQD2 (Gy**_**ab10**_**)**		
Median (range)	105.18	(42.48–123.41)
**Sum total dose in EQD2 (Gy**_**ab3**_**)**		
Median (range)	105.84	(41.47–134.78)
**Re-RT duration (days)**		
Median (range)	36	(13–69)
**Re-RT completion of planned dose**		
No	1	2.4
Yes	40	97.6
**Rest during re-RT**		
No	36	87.8
Yes	5	12.2
**Concurrent chemotherapy**		
No	20	48.8
Yes	21	51.2

*RT, radiotherapy; 3DCRT, 3D conformal radiotherapy; IMRT, intensity-modulated radiotherapy*.

For equal comparison, the total reRT dose and the sum of previous RT and reRT dose were calculated as EQD2 with an a/b value of 3 for normal tissue and 10 for tumors. All but one patient, who did not come on his own will for the last treatment session, completed the planned dose. A total of 5 patients had to rest during reRT; 1 patient did not come on his own will, 2 patients due to chemotherapy, 1 patient due to septic shock from urinary tract infection, and 1 due to perineal soreness. Thus, only 1 patient had to rest during reRT because of acute toxicity. The reason for the rest in patients who had chemotherapy was because their insurance policies did not allow chemotherapy and radiotherapy to be conducted on the same day.

### Clinical Outcomes and Toxicity After Re-Irradiation

Tumor response after reRT is shown in Supplementary Table 3. One month after reRT, 5 of the 41 cases (12.5%) showed progressive disease, and at 3 months, 3 cases (9.1%) showed progressive disease. A total of 9 patients underwent surgery after reRT. Of these, 5 patients underwent surgery due to progressive disease, while the other 4 patients underwent salvage surgery after showing stable or partial response. The pathology results showed to be Mandard grade 1, 2, or 3.

The 2-year IPFR was 49.4%, and the median IPFR was 23.3 months. Meanwhile, the 2-year OPFR was 47.9%, and the median was 22.9 months. The 2-year OS and PFS rates were 55.3 and 28.5%, respectively, and the median OS and PFS were 33.8 months and 12.8 months, respectively. Patients with recurred tumors smaller than 3.3cm showed better results for all IPFR (*p* = 0.006, Supplementary Figure 1A), OS (*p* < 0.001, Supplementary Figure 1B) and PFS (*p* = 0.020, Supplementary Figure 1C). Also, patients with tumors treated with reRT doses larger than EQD2 50 Gy_ab10_ showed better results for all IPFR (*p* < 0.001, Supplementary Figure 2A), OS (*p* = 0.002, Supplementary Figure 2B) and PFS (*p* = 0.001, Supplementary Figure 2C).

Acute and late toxicity events are shown in Table 3. For acute toxicity, 3 patients experienced grade 3 diarrhea, but there were no grade 4 or higher toxicity events. Also, no patient experienced any proctitis or cystitis as severe acute toxicity events. As for late toxicity, severe late toxicity events occurred in 17 patients, and the median time from re-irradiation to the development of severe late toxicity event was 10.5 months (range 2.3–33.3 months). A total of 8 severe fistula events (19.5%), 9 bowel obstruction events (22.0%), and 4 genitourinary toxicity events (9.8%) including ureteral or urethral stricture and hematuria occurred. Because abscess is not included in the CTCAE, abscess cases were not graded, but it was seen in 11 patients.

**Table 3:** Acute and late toxicities.

			**Severity** ***n*** **(%)**		
**Adverse event**	**None *n* (%)**	**G1**	**G2**	**G3**	**G4**
**Acute toxicity**					
Diarrhea	33 (80.5%)	3 (7.3%)	2 (4.9%)	3 (7.3%)	0 (0.0%)
Proctitis	33 (80.5%)	5 (12.2%)	3 (7.3%)	0 (0.0%)	0 (0.0%)
Cystitis	34 (82.9%)	4 (9.8%)	3 (7.3%)	0 (0.0%)	0 (0.0%)
**Late toxicity**					
Fistula	27 (65.9%)	3 (7.3%)	3 (7.3%)	7 (17.1%)	1 (2.4%)
Bowel obstruction	31 (75.6%)	1 (2.4%)	0 (0.0%)	7 (17.1%)	2 (4.9%)
GU toxicity	36 (87.8%)	0 (0.0%)	1 (2.4%)	4 (9.8%)	0 (0.0%)
	**None** ***n*** **(%)**		**Yes** ***n*** **(%)**		
Abscess	30 (73.2%)		11 (26.8%)		

*G, grade; GU, genitourinary*.

Comparison of the tumor and treatment characteristics at recurrence between those who did and did not experience severe late toxicity events showed that the tumor location was significantly different between the two groups, with a higher number of recurred tumors at the posterior or lateral location in those who did not experience severe late toxicity events (Supplementary Table 4). The median SLTFR was 33.3 months, and the 2-year SLTFR was 55.5%.

### Evaluation of Severe Late Toxicity Cases: Relationship Between the Area of Severe Late Toxicity Event and the Target Volume

ReRT plans and imaging studies at the time of severe late toxicity event were reviewed. In 3 patients, evaluation via imaging studies was not possible because the area of severe late toxicity was not clear by imaging studies. In 7 patients, reRT was performed at the gross tumor volume (GTV) with a minimal margin, and close OARs were considered when planning. In the other 7 patients, as shown in an example in Supplementary Figure 3, the related area of severe toxicity of the OAR was included in the PTV, and not the GTV.

### Analysis of Groups Divided by Treatment Aim

For additional analysis, patients were divided into three groups depending on treatment aim: group 1, salvage reRT with a surgical component (*n* = 8); group 2, definitive reRT (*n* = 23); group 3, palliative reRT (*n* = 10). Median recurred tumor size was 3.4, 2.9 and 6.0 cm for group 1, 2 and 3, respectively. Median reRT total dose was 50.0, 50.4 and 45.0 Gy and median reRT dose in EQD2 was 49.8, 53.1 and 47.1 Gy_ab10_ respectively, for group 1, 2, and 3.

**Figure 1:**
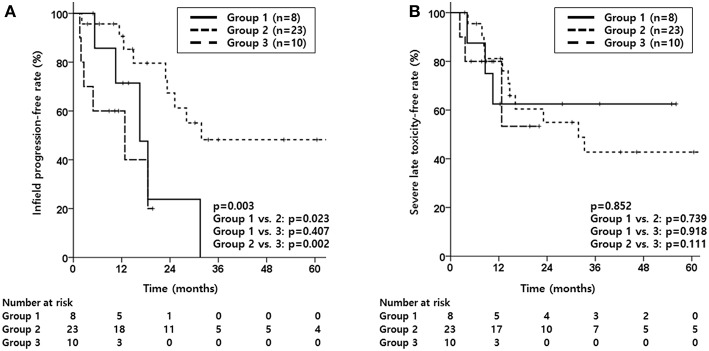
Kaplan-Meier estimates of infield progression-free rate **(A)** and severe late toxicity-free rate **(B)** according to groups divided by treatment aim (Group 1, salvage reRT with a surgical component; Group 2, definitive reRT; Group 3, palliative reRT).

Median infield progression-free period was 16.5, 31.8 and 12.8 months for group 1, 2 and 3, respectively, with group 2 showing significantly better IPFR compared to the other two groups (*p* = 0.003, Figure 1A). The OPFR showed no significant difference between the three groups (*p* = 0.243). Median OS was 33.9, 47.3, and 14.9 months for group 1, 2, and 3, respectively, with group 3 showing significantly inferior OS compared to the other two groups (*p* = 0.002, Supplementary Figure 4A). As for PFS, the median period was 10.5, 17.3, and 2.8 months, respectively, for group 1, 2, and 3, with group 3 showing significantly worse PFS compared to group 2 (*p* = 0.005, Supplementary Figure 4B).

As for toxicity, although SLTFR were not significantly different between the three groups (*p* = 0.852, Figure 1B), majority of patients who experienced severe late toxicity were in group 2 as shown in Table 4. As for those who experienced abscess, half of the patients were in group 1.

**Table 4:** Late toxicity for groups according to treatment aim.

	**Severity** ***n*** **(%)**		
**Adverse event**	**Group 1**	**Group 2**	**Group 3**
**Late toxicity**			
Fistula
Grade 0	5 (18.5%)	16 (59.3%)	6 (22.2%)
Grade 1–2	1 (16.7%)	3 (50.0%)	2 (33.3%)
Grade 3–4	2 (25.0%)	4 (50.0%)	2 (25.0%)
**Bowel obstruction**			
Grade 0	7 (22.6%)	16 (51.6%)	8 (25.8%)
Grade 1–2	0 (0.0%)	1 (100.0%)	0 (0.0%)
Grade 3–4	1 (11.1%)	6 (66.7%)	2 (22.2%)
**GU toxicity**			
Grade 0	8 (22.2%)	19 (52.8%)	9 (25.0%)
Grade 1–2	0 (0.0%)	0 (0.0%)	1 (100.0%)
Grade 3–4	0 (0.0%)	4 (100.0%)	0 (0.0%)
	Group 1	Group 2	Group 3
**Abscess**			
No	3 (10.0%)	20 (66.7%)	7 (23.3%)
Yes	5 (45.5%)	3 (27.3%)	3 (27.3%)

*G, grade; GU, genitourinary*.

### Subgroup Analysis Based on Severe Late Toxicity and Infield Progression

**Figure 2:**
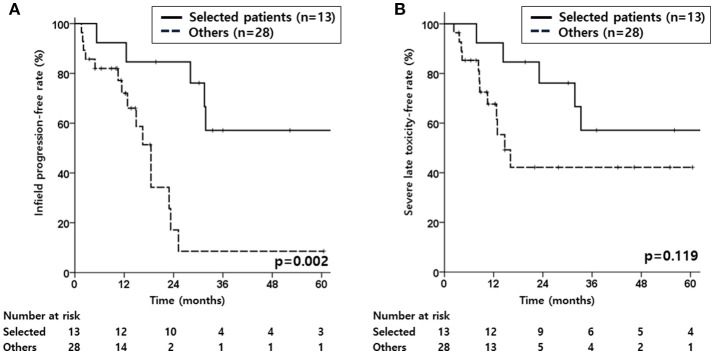
Kaplan-Meier estimates of infield progression-free rate **(A)**, and severe late toxicity-free rate **(B)** according to the selected patient subgroup or other patients.

Given that severe late toxicity events occurred in 17 patients (41.5%), a subgroup analysis was performed to investigate if there would be a group in which infield progression could be minimized with lower severe late toxicity. Considering the significant factors for IPFR, a subgroup of patients with recurred tumor size <3.3 cm and those treated with a total reRT dose of >50 Gy_ab10_ (*n* = 13) was compared with the rest of the patients. The selected subgroup showed superior IPFR (*p* = 0.002, Figure 2A), OS (*p* < 0.001), and PFS (*p* = 0.002) compared to the other patients. For SLTFR (Figure 2B), the 2-year rates were higher in the subgroup than that in the other patients (76.2 vs. 42.2%), but the difference was not statistically significant.

## Discussion

Despite the advances in treatment modalities in rectal cancer, local recurrence remains a treatment challenge for all clinicians. Although surgery is the first treatment option for patients with local recurrence, there are many cases in which reRT is needed to increase the probability of R0 resection or for inoperable cases. In our study, a total of 41 patients who received reRT to the pelvis for locoregionally recurrent rectal cancer were analyzed. The 2-year IPFR and SLTFR were 49.4 and 55.5%, respectively, with a median period of 23.3 and 33.3 months, respectively. A subgroup analysis of patients with recurred tumor size <3.3 cm and those treated with a total reRT dose of >50 Gyab_10_ showed that these factors may be used to select patients who will benefit optimally from reRT.

In our study, the median OS of 33.8 months for those who did not underwent surgery after reRT were superior compared to that in previous studies, while the median OS of 27.7 months for those who underwent surgery was inferior to that in other reports (10, 12, 13, 21). This is probably because 5 of the 9 patients who underwent surgery showed progressive disease at time of the surgery. Recently, Guren et al. reviewed the results of 10 previous studies (4 prospective studies and 6 retrospective reports) and reported a median total dose of 30 to 40 Gy to the GTV with a 1–4 cm margin and a median survival of 39–60 months and 12–16 months in patients who underwent resection and palliation, respectively, (9). Many studies reported favorable outcomes in patients treated with reRT with a salvage intent, not palliative intent (10, 12, 14, 22). In conjunction with previous studies, our additional analysis performed with groups divided by treatment aim showed the worst results for OS and PFS in group 3 (palliative reRT group).

As for acute toxicity, the rate ranged from 9 to 20% in previous studies (7, 9–14). Similarly, our study showed 3 cases of acute toxicity (7.3%), and almost all patients completed reRT without any treatment break, indicating that reRT treatment itself is tolerable. However, severe late toxicity events occurred in 17 patients (41.5%). Previously, late toxicity has been insufficiently reported, and the rate of severe late toxicity varied widely (7, 9–14, 23). In our study, we observed 7 cases of severe toxicity in which the related area of the OAR was included in the PTV. Using modern techniques such as IMRT to target only the GTV and further reducing the dose to nearby OARs may help reduce the severe late toxicity events. Additional analysis for groups divided by treatment aim showed that the majority of patients who experienced severe late toxicity were in group 2 (definitive reRT group). Group 2 may have shown the highest severe late toxicity rate since patients in group 1 may have received surgery before severe late toxicity events occurred near the recurred tumor area. Also, since group 3 showed the shortest median OS period of 14.9 months, some patients in group 3 may have expired before the occurrence of late toxicity events.

We wanted to identify a subgroup that could achieve high local control with low severe late toxicity rates. A subgroup of patients with recurred tumor size <3.3 cm and treated with a total reRT dose of >50 Gyab_10_ was compared with the other patients. Although the differences were not statistically significant for SLTFR, the findings showed that these factors are promising for properly selecting and treating patients who will optimally benefit from reRT. As for size of the recurred tumor and the total reRT dose, a phase II study by Cai et al. have attributed their low local control and clinical response rate to the large portion of patients with tumors larger than 3 cm and those who received a low total reRT dose (24). In our study, tumor size and higher reRT dose were significant factors for IPFR. Lower reRT dose maybe effective in symptom palliation, but if the tumor progresses, it will eventually cause symptoms and further re-reRT will be very challenging. In our subgroup analysis, group 2 (definitive reRT group) showed the highest IPFR compared to those treated with salvage reRT with a surgical component or palliative reRT. This may be explained by the fact than group 2 showed the smallest median recurred tumor size of 2.9 cm and highest median reRT total dose of 53.1 Gyab_10_. As for severe late toxicity, patients who did not experience severe late toxicity events showed a higher number of recurred tumors at the posterior or lateral location. In our study, the locations of the recurrent rectal tumors were classified as axial, anterior, posterior, or lateral. Because axial and anterior tumors are closer to normal organs such as the bowel or bladder, they are at a higher risk for toxicity from reRT than posterior or lateral tumors. Also, axial and anterior tumors are associated with a higher rate of R0 resection, whereas extensive posterior or lateral tumors involving the sacral promontory, iliac vessels, or the pelvic wall are contraindications for radical surgery (25, 26). Thus, location is also an important factor in deciding reRT for recurrent rectal cancer. Therefore, treating patients with recurred tumors located posteriorly or laterally and tumor size <3.3 cm with a total reRT dose of >50 Gyab_10_ to the GTV maybe the most optimal strategy.

To reduce toxicity and increase treatment efficacy for reRT, more advanced RT techniques should be developed. Recently, particle therapy such as carbon-ion therapy has gained attention due to its physical and biologic advantages (27). In Japan, the Japan Carbon-ion Radiation Oncology Study Group reported a 3-year OS rate of 73% and grade 3 late toxicity rate of 5.4% in patients with locally recurrent rectal cancer, including both reRT and non-reRT patients (28). In Germany, the first results for 19 patients with locally recurrent rectal cancer who received reRT using carbon-ion therapy were reported. Moreover, there is an ongoing phase I/II trial evaluating carbon ion radiotherapy for the treatment of recurrent rectal cancer (PANDORA-01 trial) (29, 30).

There are several limitations to this study due to its retrospective nature. First is the heterogeneity of tumor and treatment characteristics at recurrence. Second, there may have been late toxicity events in the patients lost to follow-up. Also, the small number of patients could have influenced the results. However, the number of patients is not too small compared to that of other previous studies on reRT for recurrent rectal cancer. Moreover, to the best of our knowledge, this is the first study to identify and analyze a patient subgroup that could optimally benefit from reRT.

In conclusion, our findings demonstrate that reRT for patients with locoregionally recurrent rectal cancer is a reasonable treatment option, considering the good treatment outcome and low acute toxicity rates. However, because severe late toxicity rates were substantially high, patients for reRT should be selected carefully. To improve the therapeutic ratio, reRT with curative dose for patients selected properly according to tumor size and location may be considered an attractive strategy for loco-regionally recurrent rectal cancer patients.

## Ethics Statement

This study was carried out in accordance with the recommendations of institutional review board. Written informed consent was exempted because this study was a retrospective study.

## Author Contributions

SC and HY: conceptualization and methodology. SC: validation, formal analysis, writing—original draft and visualization. SC, WK, KK, SS, JA, BM, KL, and NK: investigation and resources. SC, WK, and JC: data curation. HY: supervision.

### Conflict of Interest Statement

The authors declare that the research was conducted in the absence of any commercial or financial relationships that could be construed as a potential conflict of interest.

## Supplementary Material

Supplementary Figure 1 Kaplan-Meier estimates of infield progression-free rate **(A)**, overall survival **(B)**, and progression-free survival **(C)** according to groups divided by size.

Supplementary Figure 2 Kaplan-Meier estimates of infield progression-free rate **(A)**, overall survival **(B)**, and progression-free survival **(C)** according to groups divided by reRT dose.

Supplementary Figure 3 Sample radiotherapy plan for a patient who experienced severe late toxicity related to the organ at risk included in the PTV. This patient developed a fistulous connection between the posterior wall of the bladder and the dead space.

Supplementary Figure 4 Kaplan-Meier estimates of overall survival **(A)** and progression-free survival **(B)** according to the selected patient subgroup or other patients.

Supplementary Table 1 Initial patient and tumor characteristics.

Supplementary Table 2 Initial treatment characteristics.

Supplementary Table 3 Response after re-irradiation.

Supplementary Table 4 Factors related to severe late toxicity.

## Abbreviations

CCRT: concurrent chemoradiotherapy
CI: confidence interval
CTCAE: Common Terminology Criteria for Adverse Events
GTV: gross tumor volume
HR: hazard ratio
IMRT: intensity-modulated radiotherapy
IPFR: infield progression-free rate
OPFR: outfield progression-free rate
OARs: organs at risk
OS: overall survival
PFS: progression-free survival
PTV: planning target volume
reRT, re-irradation, RT: radiotherapy
SLTFR: severe late toxicity free-rate.
