# Supplementary figures and images for: Treatment Outcomes of Re-irradiation in Locoregionally Recurrent Rectal Cancer and Clinical Significance of Proper Patient Selection
Source: Front Oncol. 2019 Jun 19;9:529. doi: 10.3389/fonc.2019.00529 (PMC6593136; doi:10.3389/fonc.2019.00529)

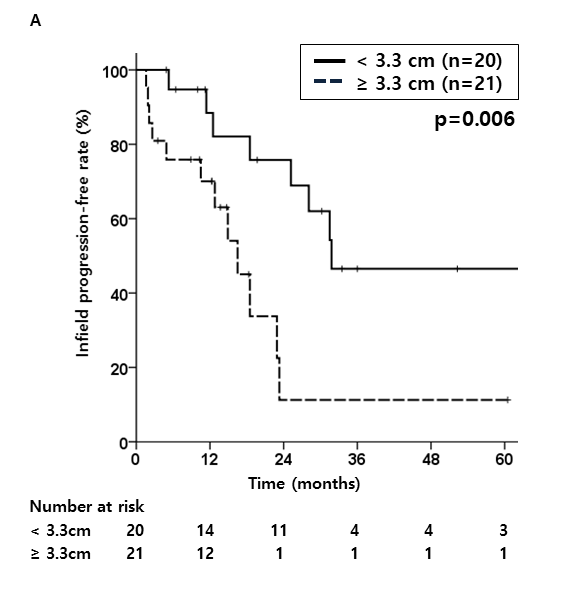

Supplement: Supplementary file 1 [file Image_1.TIF]

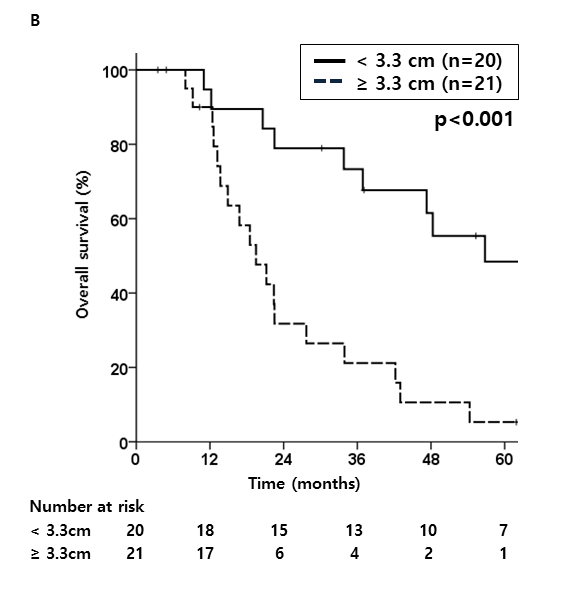

Supplement: Supplementary file 2 [file Image_2.TIF]

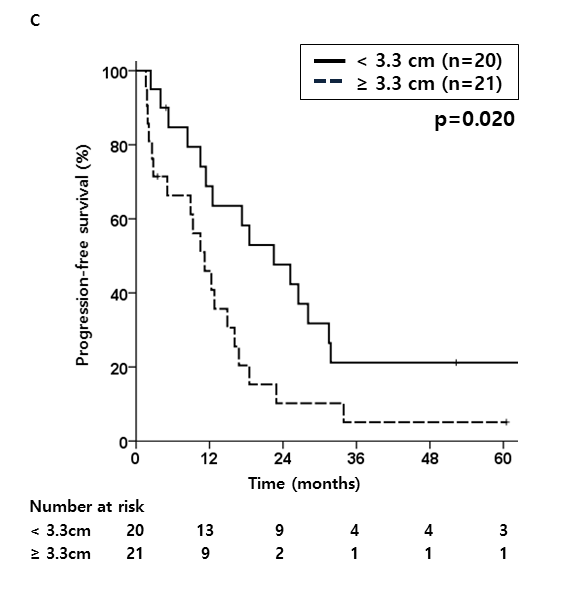

Supplement: Supplementary Figure 1 — Kaplan-Meier estimates of infield progression-free rate (A), overall survival (B), and progression-free survival (C) according to groups divided by size. [file Image_3.TIF]

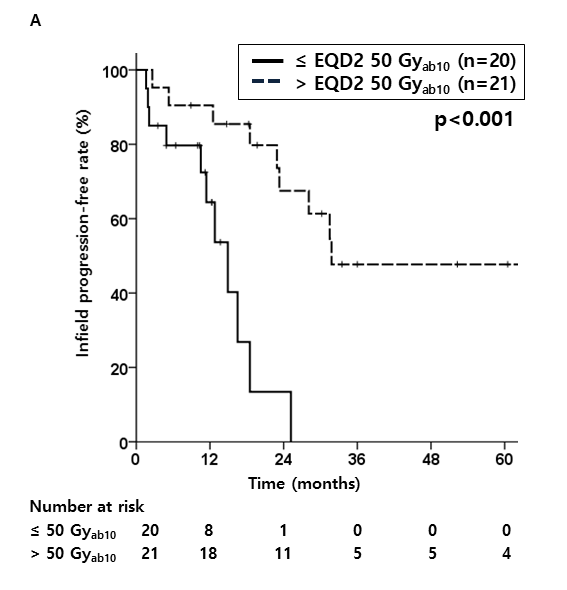

Supplement: Supplementary file 4 [file Image_4.TIF]

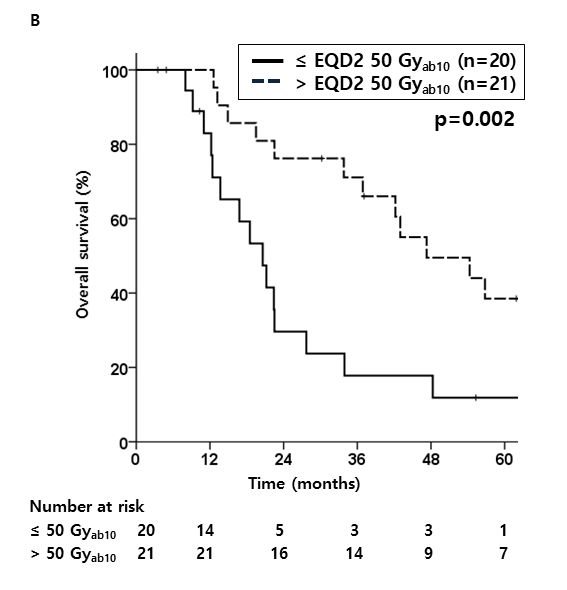

Supplement: Supplementary file 5 [file Image_5.TIF]

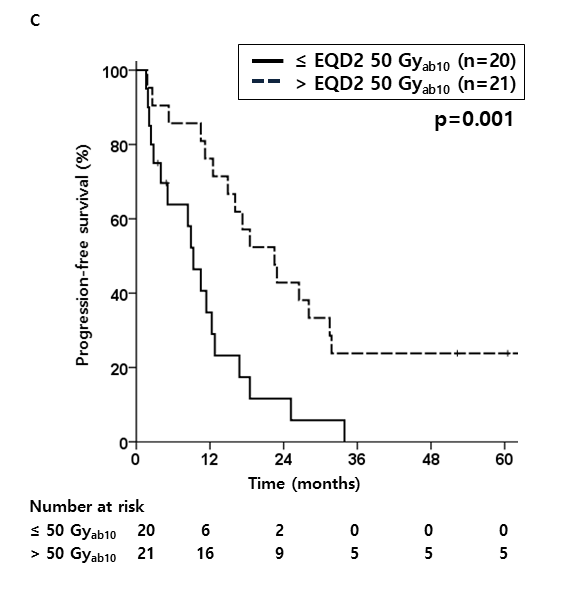

Supplement: Supplementary Figure 2 — Kaplan-Meier estimates of infield progression-free rate (A), overall survival (B), and progression-free survival (C) according to groups divided by reRT dose. [file Image_6.TIF]

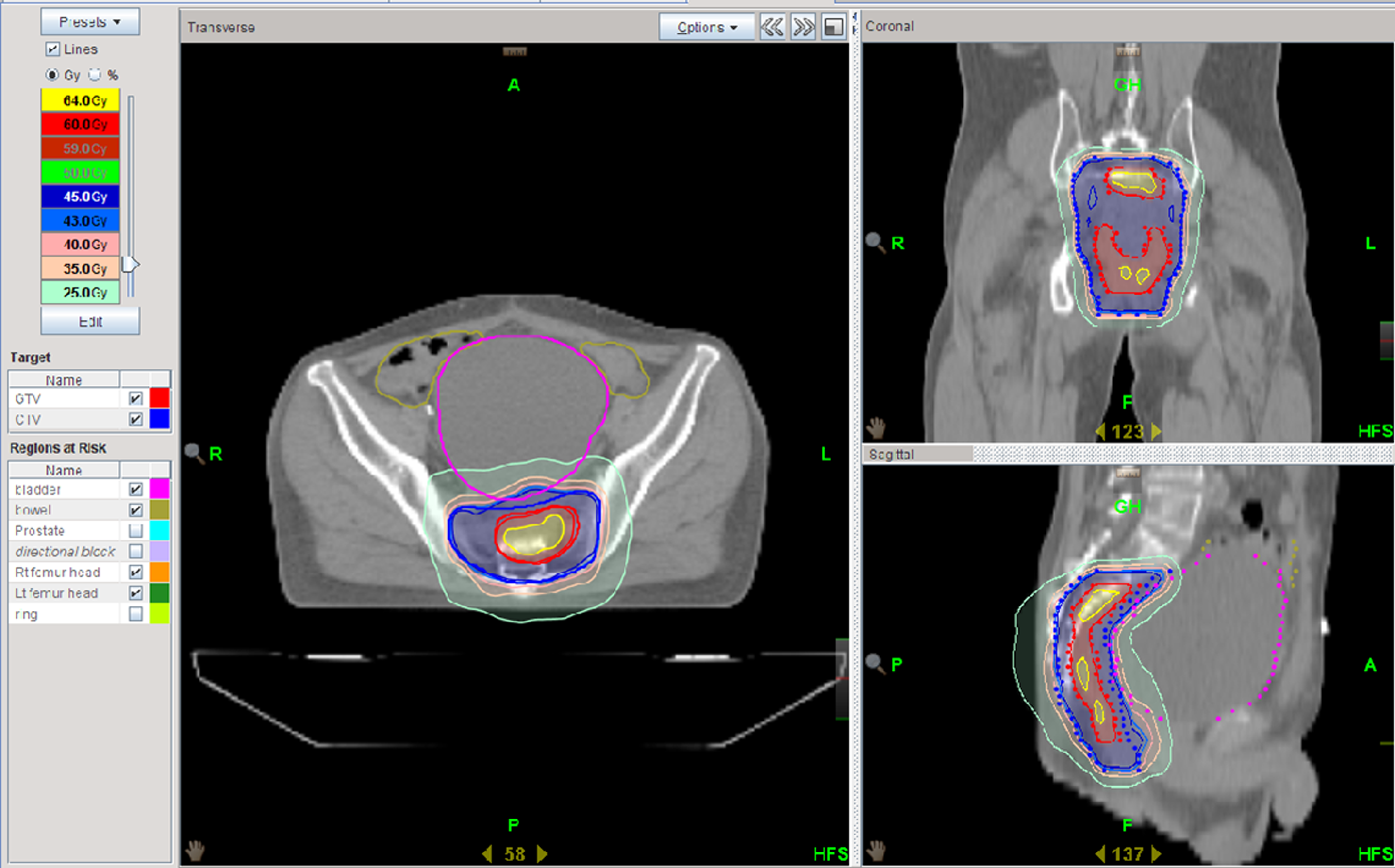

Supplement: Supplementary Figure 3 — Sample radiotherapy plan for a patient who experienced severe late toxicity related to the organ at risk included in the PTV. This patient developed a fistulous connection between the posterior wall of the bladder and the dead space. [file Image_7.TIF]

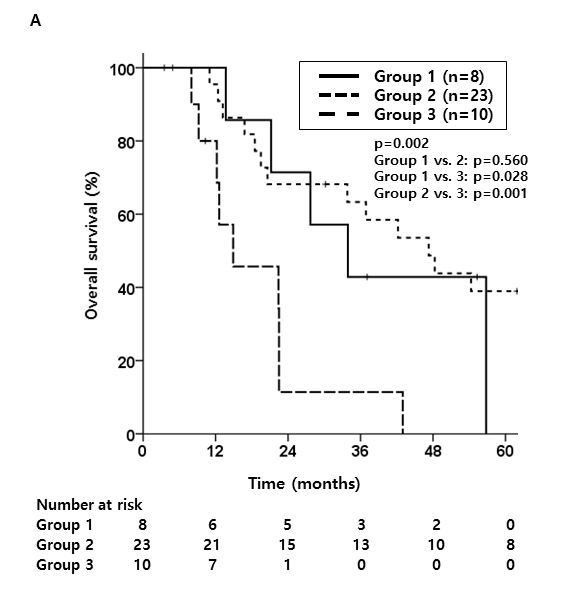

Supplement: Supplementary file 8 [file Image_8.TIF]

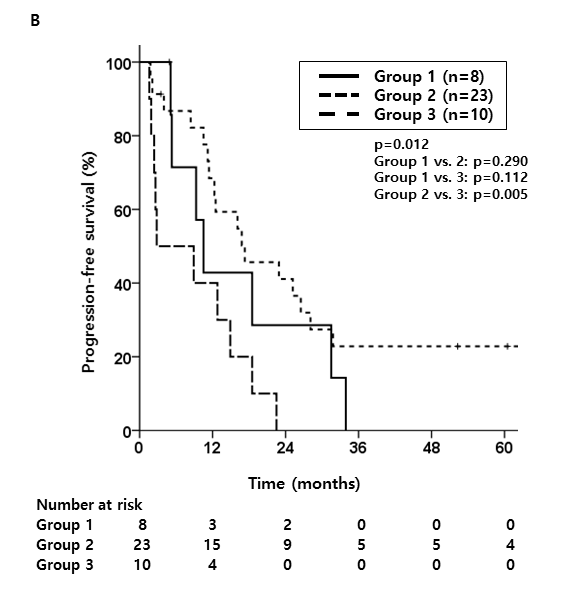

Supplement: Supplementary Figure 4 — Kaplan-Meier estimates of overall survival (A) and progression-free survival (B) according to the selected patient subgroup or other patients. [file Image_9.TIF]
