# Supplementary material for: Treatment Outcomes of Re-irradiation in Locoregionally Recurrent Rectal Cancer and Clinical Significance of Proper Patient Selection
Source: Front Oncol. 2019 Jun 19;9:529. doi: 10.3389/fonc.2019.00529 (PMC6593136; doi:10.3389/fonc.2019.00529)
Supplement: Supplementary Table 1 — Initial patient and tumor characteristics. [file Table_1.docx]

| Supplementary table 1. Initial patient and tumor characteristics | | |
| --- | --- | --- |
|  |  |  |
|  | n | % |
| Sex |  |  |
| Male | 30 | 73.2 |
| Female | 11 | 26.8 |
| Age (year) |  |  |
| Median (range) | 55 | (33–85) |
| Tumor site |  |  |
| Low | 25 | 62.5 |
| Mid | 11 | 27.5 |
| Upper | 4 | 10 |
| CRM threatening |  |  |
| Definitive | 16 | 43.2 |
| Borderline | 8 | 21.6 |
| Negative | 13 | 35.1 |
| CEA (ng/mL) |  |  |
| Median (range) | 5.5 | (0.23–85.75) |
| Pathology |  |  |
| Adenocarcinoma | 40 | 97.6 |
| Mucinous carcinoma | 1 | 2.4 |
| Pathologic grade |  |  |
| Unknown | 5 | 12.2 |
| G1 | 4 | 9.8 |
| G2 | 30 | 73.2 |
| G3 | 2 | 4.9 |
| Pathologic tumor size (cm) |  |  |
| Median (range) | 3.8 | (0.9–7.0) |
| Number of involved LNs |  |  |
| Median (range) | 1 | (0–35) |
| Resection margin |  |  |
| R0 | 32 | 78 |
| R1 | 9 | 22 |
| Pathologic T stage |  |  |
| T1 | 2 | 4.9 |
| T2 | 2 | 4.9 |
| T3 | 29 | 70.7 |
| T4 | 8 | 19.5 |
| Pathologic N stage |  |  |
| N0 | 18 | 43.9 |
| N1 | 13 | 31.7 |
| N2 | 10 | 24.4 |
| Pathologic stage |  |  |
| I | 1 | 2.4 |
| II | 16 | 39 |
| III | 24 | 58.5 |
|  |  |  |

| Supplementary table 2. Initial treatment characteristics | |  |
| --- | --- | --- |
|  |  |  |
|  | n | % |
| Radiotherapy |  |  |
| Preoperative CCRT | 21 | 51.2 |
| Postop RT | 20 | 48.8 |
| RT modality |  |  |
| 3DCRT | 39 | 95.1 |
| IMRT | 2 | 4.9 |
| Initial RT total dose (Gy) |  |  |
| Median (range) | 50.4 | (45–60) |
| Initial RT whole pelvis dose (Gy) |  |  |
| Median (range) | 45 | (41.4–50.4) |
| Initial RT dose per fraction (Gy) |  |  |
| Median (range) | 1.8 | (1.8–2.4) |
| Initial RT total dose in EQD2 (Gyab10) |  |  |
| Median (range) | 49.56 | (44.25–62) |
| Initial RT total dose in EQD2 (Gyab3) |  |  |
| Median (range) | 48.38 | (43.2–64.8) |
| Adjuvant chemotherapy |  |  |
| No | 6 | 14.6 |
| Yes | 35 | 85.4 |
| Type of surgery |  |  |
| Low anterior resection | 28 | 68.3 |
| Abdominoperineal resection | 10 | 24.4 |
| Hartmann’s procedure | 1 | 2.4 |
| Local excision | 2 | 4.9 |
| Lateral LN dissection |  | 0 |
| No | 32 | 78 |
| Yes | 9 | 22 |
| Number of dissected LNs |  |  |
| Median (range) | 16 | (1–58) |

| Supplementary table 3. Response after re-irradiation | | |
| --- | --- | --- |
|  |  |  |
|  | n | % |
| Response: post-RT 1 month |  |  |
| CR | 1 | 2.5 |
| PR | 14 | 35 |
| SD | 20 | 50 |
| PD | 5 | 12.5 |
| Response: post-RT 3 months |  |  |
| CR | 2 | 6.1 |
| PR | 10 | 30.3 |
| SD | 18 | 54.5 |
| PD | 3 | 9.1 |
| CEA: post-RT 1 month |  |  |
| Median (range) | 4.27 | (0.13–999.0) |
| CEA: post-RT 3 months |  |  |
| Median (range) | 4.5 | (0.01–999.0) |
| Surgery after reRT |  |  |
| No | 32 | 78 |
| Yes | 9 | 22 |
| Type of surgery after reRT |  |  |
| Exenteration | 2 | 22.2 |
| Abdominoperineal resection | 5 | 55.6 |
| Local excision | 2 | 22.2 |

| Supplementary table 4. Factors related to severe late toxicity | | |  |  |  |  |
| --- | --- | --- | --- | --- | --- | --- |
|  |  |  |  |  |  |  |
|  | <G3 |  |  | ≥G3 |  |  |
|  | n | % |  | n | % | p |
| Guillem class |  |  |  |  |  | 0.05 |
| Axial/anterior | 11 | 45.8 |  | 13 | 54.2 |  |
| Posterior/lateral | 13 | 76.5 |  | 4 | 23.5 |  |
| Tumor size (cm) |  |  |  |  |  | 0.654 |
| Median (range) | 3.7 | (1.5–11.0) |  | 2.5 | (1.7–11.0) |  |
| < 3.3 cm | 11 | 55 |  | 9 | 45 |  |
| ≥ 3.3 cm | 13 | 61.9 |  | 8 | 38.1 |  |
| rT stage |  |  |  |  |  | 0.79 |
| rT0-3 | 8 | 61.5 |  | 5 | 38.5 |  |
| rT4 | 16 | 57.1 |  | 12 | 42.9 |  |
| rN stage |  |  |  |  |  | 1.000* |
| rN0 | 17 | 58.6 |  | 12 | 41.4 |  |
| rN1-2 | 7 | 60 |  | 5 | 40 |  |
| Concurrent chemotherapy |  |  |  |  |  | 0.853 |
| No | 12 | 60.0 |  | 8 | 40.0 |  |
| Yes | 12 | 57.1 |  | 9 | 42.9 |  |
| RT modality |  |  |  |  |  | 0.885 |
| 3DCRT | 9 | 60 |  | 6 | 40 |  |
| IMRT/Cyberknife | 15 | 57.7 |  | 11 | 42.3 |  |
| Surgery after reRT |  |  |  |  |  | 0.128* |
| No | 21 | 65.6 |  | 11 | 34.4 |  |
| Yes | 3 | 33.3 |  | 6 | 66.7 |  |
| Re-RT total dose (Gy) |  |  |  |  |  | 0.812 |
| Median (range) | 50 | (30.0–60.0) |  | 50.4 (30.6–60.0) | |  |
| ≤ 50.4 Gy | 15 | 60 |  | 10 | 40 |  |
| > 50.4 Gy | 9 | 56.3 |  | 7 | 43.8 |  |
| Re-RT TD in EQD2 (Gyab3) |  |  |  |  |  | 0.476 |
| Median (range) | 55 | (25.2–86.4) |  | 50.0 (29.4–72.0) | |  |
| ≤ 50 Gy | 10 | 52.6 |  | 9 | 47.4 |  |
| > 50 Gy | 14 | 63.6 |  | 8 | 36.4 |  |
| Sum total dose (Gy) |  |  |  |  |  | 0.326 |
| Median (range) | 102.3 | (81.0–119.4) |  | 106.8 (43.2–119.4) | |  |
| ≤ 105 Gy | 15 | 65.2 |  | 8 | 34.8 |  |
| > 105 Gy | 9 | 50 |  | 9 | 50 |  |
| Sum TD in EQD2 (Gyab3) |  |  |  |  |  | 0.654 |
| Median (range) | 106.3 | (77.0–134.8) |  | 103.7 (41.5–129.0) | |  |
| ≤ 105 Gy | 11 | 55 |  | 9 | 45 |  |
| > 105 Gy | 13 | 61.9 |  | 8 | 38.1 |  |
| Time interval between initial RT to reRT |  |  |  |  |  | 0.975 |
| Median (range) | 25.6 | (6.6–138.9) |  | 26.0 (4.5–73.2) | |  |
| ≤ 25 months | 10 | 58.8 |  | 7 | 41.2 |  |
| > 25 months | 14 | 58.3 |  | 10 | 41.7 |  |
